# Neuroglobin in Breast Cancer Cells: Effect of Hypoxia and Oxidative Stress on Protein Level, Localization, and Anti-Apoptotic Function

**DOI:** 10.1371/journal.pone.0154959

**Published:** 2016-05-05

**Authors:** Marco Fiocchetti, Manuela Cipolletti, Stefano Leone, Antonella Naldini, Fabio Carraro, Daniela Giordano, Cinzia Verde, Paolo Ascenzi, Maria Marino

**Affiliations:** 1 Department of Science, Roma Tre University, Viale Guglielmo Marconi 446, I-00146 Roma, Italy; 2 Department of Molecular and Developmental Medicine, University of Siena, Via Aldo Moro 2, 53100 Siena, Italy; 3 Biosciences and BioResources Institute—CNR, Via Pietro Castellino 111, 80131 Napoli, Italy; 4 Interdepartmental Laboratory of Electron Microscopy, Roma Tre University, Via della Vasca Navale 79, I-00146 Roma, Italy; National Institutes of Health, UNITED STATES

## Abstract

The over-expression of human neuroglobin (NGB), a heme-protein preferentially expressed in the brain, displays anti-apoptotic effects against hypoxic/ischemic and oxidative stresses enhancing neuron survival. As hypoxic and oxidative stress injury frequently occurs in fast proliferating neoplastic tissues, here, the effect of these stressors on the level, localization, and anti-apoptotic function of NGB in wild type and NGB-stable-silenced MCF-7 breast cancer cells has been assessed. The well-known endogenous NGB inducer 17β-estradiol (E2) has been used as positive control. The median pO2 present in tumor microenvironment of breast cancer patients (*i*.*e*., 2% O_2_) does not affect the NGB level in breast cancer cells, whereas hydrogen peroxide and lead(IV) acetate, which increase intracellular reactive oxygen species (ROS) level, enhance the NGB levels outside the mitochondria and still activate apoptosis. However, E2-induced NGB up-regulation in mitochondria completely reverse lead(IV) acetate-induced PARP cleavage. These results indicate that the NGB level could represent a marker of oxidative-stress in MCF-7 breast cancer cells; however, the NGB ability to respond to injuring stimuli by preventing apoptosis requires its re-allocation into the mitochondria. As a whole, present data might lead to a new direction in understanding NGB function in cancer opening new avenues for the therapeutic intervention.

## Introduction

Neuroglobin (NGB) is a relatively recent discovered monomeric heme-protein so named because of its preferential expression in the nervous system [1]. NGB over-expression, driven by transiently transfected pcDNA vector, protects cultured neurons against hypoxia [2], enhances neuron survival under anoxia or oxygen/glucose deprivation [3], and displays neuro-protective properties against hypoxic/ischemic and oxidative stress [4–7]. In addition, in brain-derived cell lines, hypoxia [8], H_2_O_2_ injury [9], and lipopolysaccharides [10] moderately induce NGB. This suggests that NGB could behave in neurons as a sensor of injuring stimuli including oxidative stress, hypoxia, and neurotoxicity. However, as described in detail previously, no differences in NGB levels in murine models of traumatic brain injury, experimental autoimmune encephalitis, cerebral malaria, and hypoxia have been found [11–14], thus insinuating uncertainty on the role of NGB as a stress sensor.

Mechanism(s) by which NGB over-expression exerts its effect in neurons are uncertain. In particular, NGB has been postulated to enhance O_2_ supply, to scavenge reactive oxygen species (ROS) [13, 15], and to modulate nitric oxide homeostasis in active neurons [8, 16]. Moreover, oxidative stress (*i*.*e*., H_2_O_2_ treatment) induces the specific binding of transiently over-expressed NGB to flotillin-1, a protein associated to the plasma membrane lipid raft micro-domains, leading to cell survival [17]. Furthermore, the sex steroid hormone 17β-estradiol (E2) increases the endogenous level of NGB in several brain-derived cells and induces the heme-protein reallocation into mitochondria where, upon oxidative stress injury, it interacts with mitochondrial cytochrome *c* avoiding its release into the cytosol and the activation of the apoptotic cascade [9, 10]. Whether mitochondrial and/or cytosolic NGB mechanisms act synergistically or antagonistically in neurons to respond to oxidative stress or hypoxia is unknown.

Besides neuronal damage, oxidative stress and hypoxia are conditions frequently occurring in fast proliferating neoplastic tissues. Indeed, cancer cells adapt themselves to the stressful and dynamic microenvironment of solid tumors, where the redox status is imbalanced and oxygen/nutrients availability is limited [18, 19]. The adaptation is achieved by developing alternative compensatory metabolic reactions that render cancer cells insensitive to stress inducers such as chemotherapy and radiation [19]. Although a tumor suppressive function of transiently over-expressed NGB in hepatoma cancer cells has been described [20], other studies reported that NGB expression is differentially modulated by hypoxia and oxidative stress in cancer cell lines [21, 22]. This suggests that NGB may be part of the defense mechanism established by cancer cells to counteract tumor environment stress condition by helping cells to survive [21, 22]. In line with these last studies, it has been demonstrated that NGB up-regulation is one of the vital mechanisms triggered by E2 to increase the cell survival by preventing the apoptotic cascade of E2-dependent cancer cells (breast, hepatoma, and colon cancer cell lines) in the presence of oxidative stress [23, 24]. As a whole, these results suggest that NGB could act in cancer cells, like in neurons, as a compensatory protective protein activated in response to injuring stimuli and able to prevent mitochondria-dependent apoptosis. To evaluate this hypothesis the effect of hypoxia, hydrogen peroxide (H_2_O_2_), and lead(IV) acetate (Pb(IV)) on the level, localization, and function of NGB in wild-type and NGB stable silenced MCF-7 breast cancer cells has been assessed.

## Materials and Methods

### Reagents

E2, actinomycin D (Act), Pen-Strep solution, H_2_O_2_, RPMI-1640 media without phenol red, Dulbecco’s modified Eagle medium (DMEM) without phenol red, charcoal-stripped fetal calf serum, protease inhibitor cocktail, bovine serum albumin fraction V (BSA), 2’,7’-dichlorofluorescin diacetate (DCFH-DA), puromycin, staurosporine, and Pb(IV), were purchased from Sigma-Aldrich (St. Louis, MO, USA). The translational inhibitor, Cicloheximide (Ciclohex), was purchased by Tocris (Tocris Bioscience, Italy). Bradford protein assay was obtained from Bio-Rad Laboratories (Hercules, CA, USA). Short hairpin RNA (shRNA) of NGB Lentiviral Particles, Control shRNA Lentiviral Particles, anti-poly(ADP ribose) polymerase (PARP-1), anti-NGB, anti-Bcl2 antibodies and Annexin V-FITC Apoptosis Detection Kit were obtained from Santa Cruz Biotechnology (Santa Cruz, CA, USA). The chemiluminescence reagent for Western blot super power ECL was obtained from Bio-Rad (Milan, Italy). All the other products were from Sigma-Aldrich. Analytical or reagent grade products were used without further purification.

### Preparation and purification of human recombinant NGB

NGB cDNA was cloned into the pET3a vector (Novagen EMD Biosciences, Inc., Madison, WI, USA). The overexpression of NGB was induced in the *Escherichia coli* strain BL21(DE3)pLysS (Invitrogen, Carlsbad, California, USA) by treatment with 0.4 mM of isopropyl-D-thiogalactopyranoside (IPTG) in the presence of the heme-precursor aminolevulinic acid (1 mM). Soluble cell extract was loaded onto a DEAE-Sepharose Fast Flow (GE Healthcare Biosciences, Amersham Biosciences Ltd, UK) anion-exchange column equilibrated with 5 mM Tris-HCl, pH 8.5 and fractions were eluted with a NaCl gradient (from 0 to 300 mM). Eluted NGB was further purified by passage through a Sephacryl S-100 (GE Healthcare Biosciences, Amersham Biosciences Ltd, UK) gel filtration column. The protein obtained was > 98% pure on SDS-PAGE. The NGB concentration was determined spectrophotometrically, acquiring UV-visible spectra on a Cary 300 spectrophotometer (Varian, Palo Alto, CA). Five ng of recombinant NGB (final dilution: 1μg/1μl) were loaded in Western blot and the intensity of the bands was compared by densitometric analyses (see below). Note that, due to recombinant NGB purification, its migration on SDS PAGE resulted faster than that of NGB present in whole cell lysates.

### Cell culture

Human breast cancer cells MCF-7 (ATTC, LGC Standards S.r.l., Milano, Italy) were routinely grown in air containing 5% CO_2_ in modified, phenol red-free, DMEM medium containing 10% (v/v) charcoal-stripped fetal calf serum, L-glutamine (2 mM), gentamicin (0.1 mg/ml) and penicillin (100 U/ml). Cells were passaged every 2 days and media changed every 2 days. The cell lines were grown as previously described [23] and used at passage 4–8. The cell line authentication was periodically performed by amplification of multiple STR loci by BMR genomics srl (Padova, Italy). NGB stably-silenced MCF-7 cells were obtained with short hairpin RNA (shRNA) lentivirus particles (Santa Cruz, CA, USA) as previously described [23]. The lentiviral infected MCF-7 cell line was routinely grown in media containing puromycin (0.5 μg/ml). Cells were treated for 24 h with either vehicle (ethanol/PBS 1:10, v/v) or E2 (10 nM) or H_2_O_2_ (400 μM) or Pb(IV) (200μM). Cells were harvested with trypsin and centrifuged 24 h after treatment.

### Hypoxic treatment

MCF-7 cell lines were grown to 70% confluence in 6-well plates and stimulated with either vehicle or E2 (10 nM). After 2h of stimulation, cells were cultured in normoxia using an incubator (KW Apparecchi Scientifici, Siena, Italy) set at 5% CO_2_, 21% O_2_ (atmospheric oxygen ~140 mmHg), and 37.0°C in a humidified environment. For the experiments under hypoxia, a water-jacketed incubator (Forma Scientific, Marietta, OH, USA) has been used to provide a customized and stable humidified environment through electronic control of CO_2_ (5%), O_2_, and temperature (37.0°C). The O_2_ tension was set and maintained constantly at 2% (~14mmHg) by injecting N_2_ automatically in the chamber.

### Western blot assay

Protein extraction and Western blot assay were performed as reported elsewhere [9]. Briefly, after treatment, cells were lysed and solubilized in the sample buffer containing 0.125 M Tris-HCl, pH 6.8, and 10% (w/v) SDS [9]. Total proteins were quantified using the Bradford Protein Assay. Solubilized proteins (20 μg) were resolved by 7% or 15% SDS-PAGE at 100 V for 1 h at 24.0°C and then transferred to nitrocellulose with the Trans-Blot Turbo Transfer System (Bio-Rad, Hercules, CA) for 7 min. The nitrocellulose was treated with 3% (w/v) BSA in 138.0 mM NaCl, 25.0 mM Tris, pH 8.0, at 24.0°C for 1 h and then probed overnight at 4.0°C with either anti-NGB (final dilution 1:1000), anti-PARP-1 (final dilution 1:1000), and anti-Hypoxia-inducible factor-1α (HIF1α) (final dilution 1:1000) antibodies. The nitrocellulose was stripped by the Restore Western Blot Stripping Buffer (Pierce Chemical, Rockford, IL, USA), for 10 min at room temperature, and then probed with anti-β-tubulin antibody (final dilution 1:1000) to normalize protein loaded. The antibody reaction was visualized with the chemiluminescence Western blotting detection reagent (Amersham Biosciences, Little Chalfont, UK). The densitometric analyses were performed by ImageJ software for Microsoft Windows (National Institutes of Health, Bethesda, MD, USA).

### Intracellular ROS measurement

Cells were seeded in clear bottom 96-well microplate with 2.5×10^4^ cells per well. After allowing cells to adhere overnight, the medium was removed and cells washed once with serum-free medium. Then, cells were incubated with DCFH-DA (20 μM) at 37°C, 30 min in the dark. After this time, the DCFH-DA solution was removed and cells washed once with serum-free medium and treated with selected compounds; background wells (untreated stained cells) as well as blank wells (medium only) were included. The microplates were read in the presence of compounds and media on a multi-label plate reader (VICTOR™ X3 Multilabel Plate Reader, PerkinElmer, Waltham, MA, USA) with excitation wavelength at 485 nm and emission wavelength at 535 nm to measure fluorescence intensity for each time interval (from 0 to 6 h). The fluorescence was registered as arbitrary units, the ratio between the single treatment induced fluorescence, and the vehicle fluorescence was plotted for each time considered.

### Stress and Apoptosis Signaling Measurement

For the simultaneous detection of 19 signaling molecules that are involved in the regulation of the stress response and apoptosis, the PathScan® Stress and Apoptosis Signaling Antibody Array Kit has been used according to the manufacturer’s instructions (Cell Signaling Technology, Danvers, MA, USA). Briefly, MCF-7 cells were grown until 80% confluence, treated with the selected compounds, and lysed in 1X Cell Lysis Buffer to collect cell lysates. The array-blocking buffer was added to each well for 15 min at room temperature. Then, 30 μg of solubilized proteins were added to wells and incubated for 2 h at room temperature. Subsequently, the Detection Antibody Cocktail supplied with the kit was added and maintained for 1 h at room temperature. The slide was then incubated for 30 min with horseradish peroxidase-linked streptavidin solution at room temperature. Finally, the slide was covered with LumiGLO/Peroxide reagent (supplied with the kit) and exposed to chemiluminescence film (Amersham Biosciences, Little Chalfont, UK) for 2 to 60 sec. The images were then acquired and the signal intensity was measured using the ImageJ software for Microsoft Windows (National Institutes of Health, Bethesda, MD, USA).

### Quantitative Real-Time Polymerase Chain Reaction

The sequences for gene-specific forward and reverse primers were designed using the OligoPerfect Designer software program (Invitrogen). The following primers were used: for human NGB 5’-GTCTCTCCTCGCCTGAGTTC-3’(forward) and 5’-GACTCACCCACTGTCGAGAA -3’ (reverse) and for human GAPDH, 5’-CGAGATCCCTCCAAAATCAA-3’ (forward) and 5’-TGTGGTCATGAGTCCTTCCA-3’ (reverse). Total RNA was extracted from cells using TRIzol Reagent (Invitrogen) according to the manufacturer’s instructions. To determine NGB gene expression levels, cDNA synthesis and qPCR were performed using the GoTaq two-step RT-qPCR system (Promega) in an ABI Prism 7900HT Sequence Detection System (Applied Biosystems, Foster City, CA) according to the manufacturer’s instructions. Each sample was tested in triplicate and the experiment repeated twice. All primers used were optimized for real-time amplification in a standard curve amplification (>98% for each pair of primers) and verifying the production of a single amplicon in a melting curve assay. Results were normalized to the expression of GAPDH mRNA. The relative level for NGB gene, reported in arbitrary units, was calculated using the 2-ΔΔCt method.

### shRNA Lentiviral particles transduction

shRNA lentiviral particles transduction was performed using control shRNA Lentiviral particles (Santa Cruz sc-108080) and Neuroglobin shRNA lentiviral particles (Santa Cruz sc-42081-v) according to manufacturer’s instructions as previously described [23].

### Apoptosis measurement

Phosphatidylserine externalization was quantified by flow cytometry by using the Annexin V-FITC Apoptosis Detection Kit including propidium iodide (PI) according to the manufacturer’s guideline (Santa Cruz, CA, USA). Briefly, both attached and floating cells were collected after treatment(s), washed twice with cold PBS and re-suspended in the annexin-binding buffer at a concentration of ~1×10^6^ cells/ml; 100 μl of the cell suspension (~1×10^5^ cells) were transferred to a culture tube and 2.5 μl of annexin V-FITC and 10 μl of PI were added. After incubation in the dark (15 min at room temperature), 400 μl of the binding buffer were added and cells were analyzed immediately by flow cytometry with the DAKO Galaxy flow-cytometer equipped with HBO mercury lamp. Analysis by flow cytometry used the FL1 (FITC) and FL3 (PI) laser lines; each sample was assessed using a collection of 10,000 events. Each experiment was carried out in triplicate and the fluorescence was calculated using a FloMax^©^ Software.

### Mitochondria isolation

Cell fractionation was performed using ApoAlert™ Cell Fractionation kit (Clontech Laboratories Inc. Mountain View, CA, USA) according to manufacturer’s instructions. After stimulation, cells were harvested with trypsin (1%, v/v), suspended with complete medium, and centrifuged at 600g for 5 min. Pellet was suspended in Fractionation Buffer Mix containing DTT 1 mM and homogenized in a Dounce tissue grinder. Homogenate was centrifuged at 700g for 10 min. Pellet was suspended in Fractionation Buffer Mix to obtain mitochondrial fraction. The mitochondrial TNF-receptor associated protein 1 (TRAP-1) and cytosolic Protein Phosphatase 2A (PP2A) markers were used as mitochondrial fraction purity indicators. Protein concentration of each fraction was determined using Bradford protein assay. Lysate of each fraction was then processed for Western Blot or used for immunoprecipitation assay.

### Confocal microscopy analysis

MCF-7 cells were stained with anti-NGB (1:200) and anti-COX-4 (1:200) antibodies, respectively. Cells were processed and confocal analysis were performed as previously described [23]. The 8.2 IMARIS software was used to quantify NGB-COX-4 merged signals.

### Statistical analysis

The statistical analysis was performed by Student’s *t*-test with the INSTAT software system for Windows. In all cases, only probability (*p*) values below 0.05 were considered significant.

## Results

### Effect of hypoxia on NGB levels

**Fig 1:**
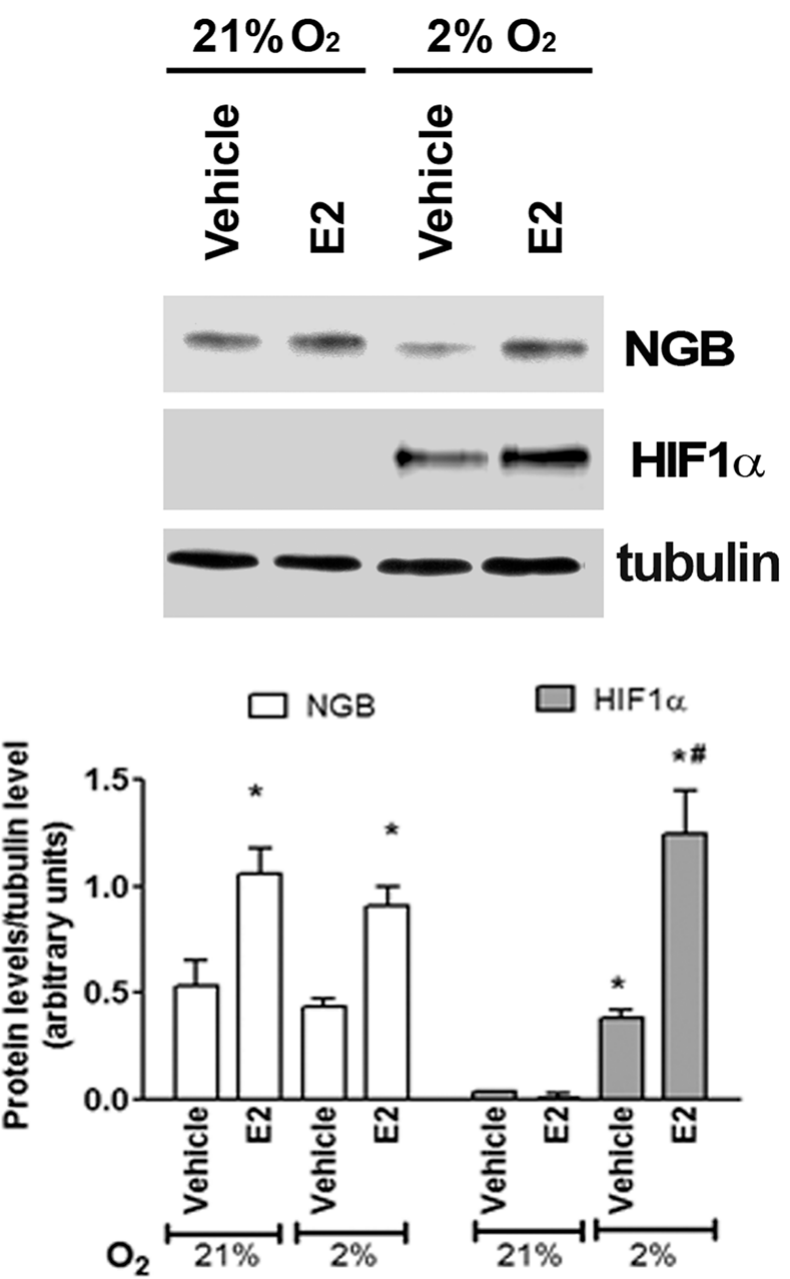
Effect of hypoxia in MCF-7 cells. NGB and HIF1α protein expression in MCF-7 cells exposed to either normoxia (21% O_2;_ 24 h) or physiological hypoxia (2% O_2_; 24 h), in the presence and absence of E2 (10 nM; 2h pretreatment). The amount of proteins were normalized to tubulin levels. Top panel is typical Western blot of three independent experiments. Bottom panel represents the results of the densitometric analysis. Data are means ± SD of three different experiments. P<0.05 was determined with Student t-test vs. normoxia vehicle (*) and vs. hypoxia vehicle (#).

Neither 24 h (Fig 1) nor 48 h (data not shown) of physiological hypoxia (2% O_2_) increases NGB protein levels in comparison to normoxia (21% O_2_) in breast cancer cells. However, E2 treatment (10 nM, 24 h) still induces the up-regulation of NGB and the hypoxia sensor HIF1α (Fig 1).

### Effect of ROS-inducing compounds on NGB levels

**Fig 2:**
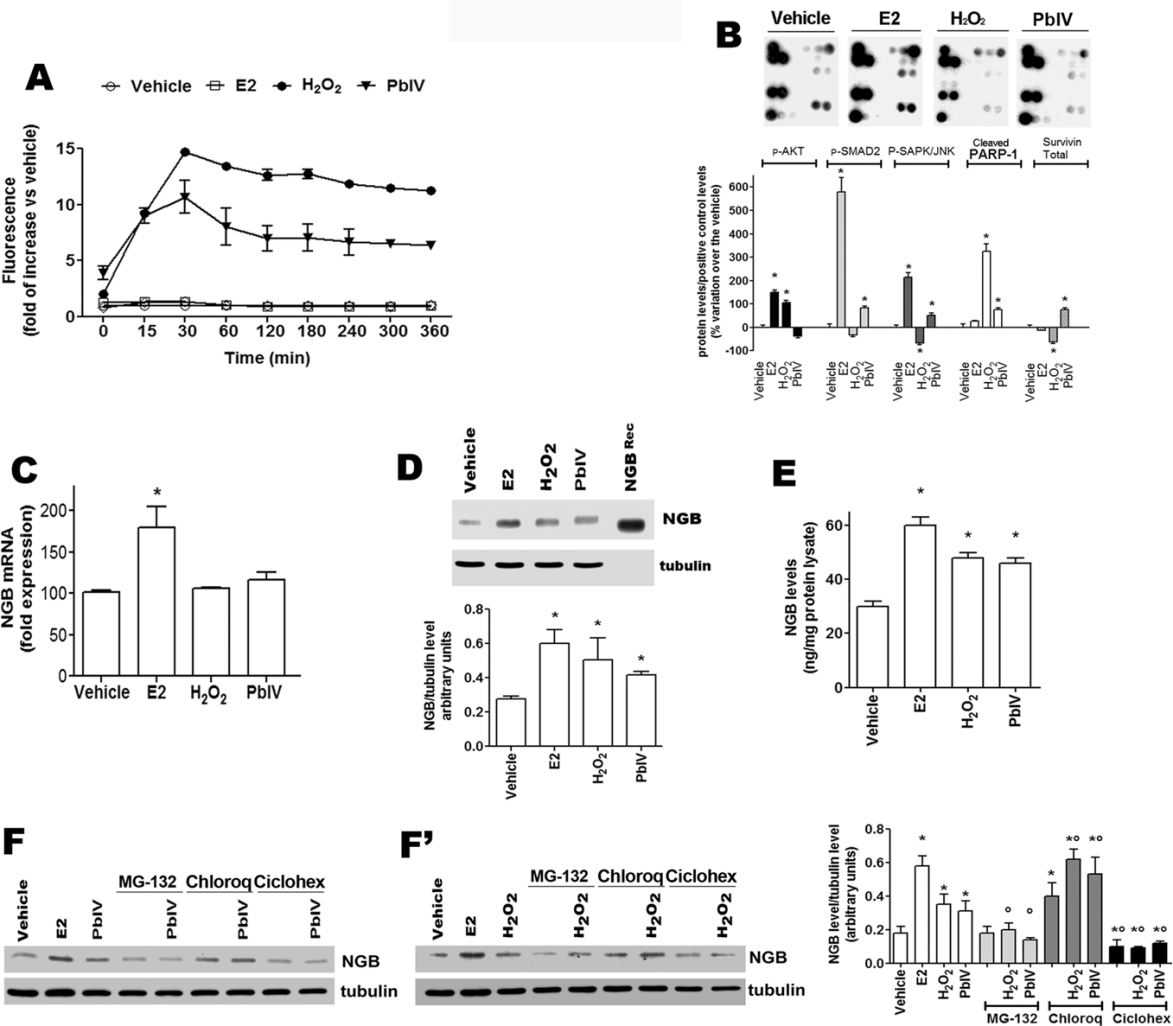
Characterization of H_2_O_2_ and Pb(IV) as MCF-7 cell stressors. **(A)** Cells were exposed to E2 (10 nM), H_2_O_2_ (400 μM), and Pb(IV) (200 μM). ROS measurement was obtained by DCFH-DA fluorescence analysis. **(B**) PathScan analysis of 19 target signaling proteins involved in the regulation of stress response and apoptosis in MCF-7 cells treated for 24 h with E2 (10 nM), H_2_O_2_ (400 μM), Pb(IV) (200 μM). Top panels are typical chemioluminescent signal of array modules of three independent experiments. Bottom panel represents the result of densitometric analysis of p-AKT, p-SMAD2, p-SAPK/JNK, cleaved PARP-1 and total Survivin proteins. Data are means ± SD of three different experiments. P< 0.05 was calculated with Student’s t test vs vehicle (*). **(C**) NGB mRNA levels in MCF-7 cells. The NGB expression is reported as fold of induction over the vehicle (set to 100). Data represent the mean ±SD of five different experiments. Significant differences (p<0.001) were determined by ANOVA followed by the Turkey-Kramer post-test with respect to unstimulated samples (*). **(D)** Analysis of NGB protein levels in cells treated with the above reported compounds for 24 h. The amount of protein was normalized to tubulin levels. Top panel is typical Western blot of three independent experiments. Bottom panel represents the result of densitometric analyses. Data are means ± SD of three different experiments. P<0.05 was determined with Student t-test vs. vehicle (*). **(E**) NGB protein amount in treated cells. NGB protein cell content was quantified by comparing the Western blot band intensity of treated sample NGB with the band intensity of 5 ng NGB recombinant protein used as protein standard. Data are means ± SD of three independent experiments. P<0.05 was determined with Student t-test vs. vehicle (*). **(F and F’**) MCF-7 cells were pre-treated for 30 min with the proteasomal inhibitor, MG-132 (1 μM), the lysosomal inhibitor, Chloroquine (Chloroq, 10 μM), and the translational inhibitor Cicloheximide (Ciclohex, 10 μM) before H_2_O_2_ (400 μM) and Pb(IV) (200 μM) treatment for 24 h. The amount of NGB was normalized to tubulin levels. Left panels are typical Western blots of three independent experiments. Right panel represents the result of densitometric analyses. Data are means ± SD of five different experiments. P<0.05 was determined with Student t-test vs. vehicle (*) or vs. non-treated samples (°).

To determine the role of NGB during oxidative stress injury, MCF-7 cells have been treated with H_2_O_2_ and Pb(IV), (a pollutant which induces oxidative stress and mitochondrial-dependent apoptosis) [25]. E2 treatment has been used as the positive control. Neither vehicle nor E2 (10 nM) enhance ROS level in MCF7 cells, whereas cell treatment with either H_2_O_2_ (400 μM) or Pb(IV) (200 μM) increased the ROS production reaching maximum levels after 30 min of treatment (14.68 ± 0.04 and 11.00 ± 1.49 fold over the control, respectively) (Fig 2A). Moreover, the E2-, H_2_O_2_-, and Pb(IV)-activated signaling pathways involved in cell response to stress and apoptosis has been evaluated by the PathScan array kit (S1 Fig). As shown in Fig 2B, E2 activates the phosphorylation of AKT, SMAD2, and SAPK/JNK in line with its well-known function as activator of MCF-7 survival, proliferation, and migration [26]. On the other hand, H_2_O_2_ enhances PARP-1 cleavage and AKT phosphorylation, impairing SAPK/JNK phosphorylation and decreasing survivin levels; while, Pb(IV) increases the phosphorylation of SMAD2 and SAPK/JNK as well as the PARP-1 cleavage and survivin level (Fig 2B). Finally, the capability of E2, H_2_O_2_, and Pb(IV) to modify the level of NGB mRNA (Fig 2C) and protein (Fig 2D, 2F and 2F’) has been evaluated. E2 induces the increase of NGB mRNA 4 h after treatment (Fig 2C), whereas neither H_2_O_2_ nor Pb(IV) modulate NGB mRNA levels (Fig 2C). Conversely, like E2, both H_2_O_2_ and Pb(IV) increase NGB protein levels (Fig 2D). To quantify the results obtained by Western blot, the intensity of the NGB bands was compared with that obtained loading 5 ng of recombinant NGB. MCF-7 cells contain a very low basal level of NGB (30 ± 3.3 ng/mg protein lysate) which significantly doubles 24 h after E2 (60 ± 3.2 ng/mg protein lysate), H_2_O_2_ (48 ± 2.2 ng/mg protein lysate), and Pb(IV) (46 ± 2.1 ng/mg protein lysate) treatment (Fig 2E). In order to obtain clear evidence how NGB level could be regulated by H_2_O_2_ and Pb(IV), MCF-7 cells were treated with either the proteasomal inhibitor, MG-132 (1 μM for 30 min), the lysosomal inhibitor, Chloroquine (Chloroq, 10 μM for 30 min), and the translational inhibitor Cicloheximide (Ciclohex, 10 μM for 30 min) before the treatment with the ROS-inducers. Fig 2F and 2F’ show that NGB level is reduced by ciclohex and increased by lysosomal degradation. H_2_O_2_ and Pb(IV) treatments do not modify this trend suggesting that ROS-inducing compounds could increase NGB levels by inhibiting lysosomal degradation and increasing NGB translation. Intriguingly, MG-132 does not modulate NGB level, but completely impairs H_2_O_2_ and Pb(IV) effect in enhancing NGB level (Fig 2F and 2F’). As a whole, these data indicate that ROS-inducing compounds increase NGB protein levels in MCF-7 cancer cells activating specific and diverse pathways.

### Effect of H_2_O_2_ and Pb(IV) on mitochondrial NGB localization

**Fig 3:**
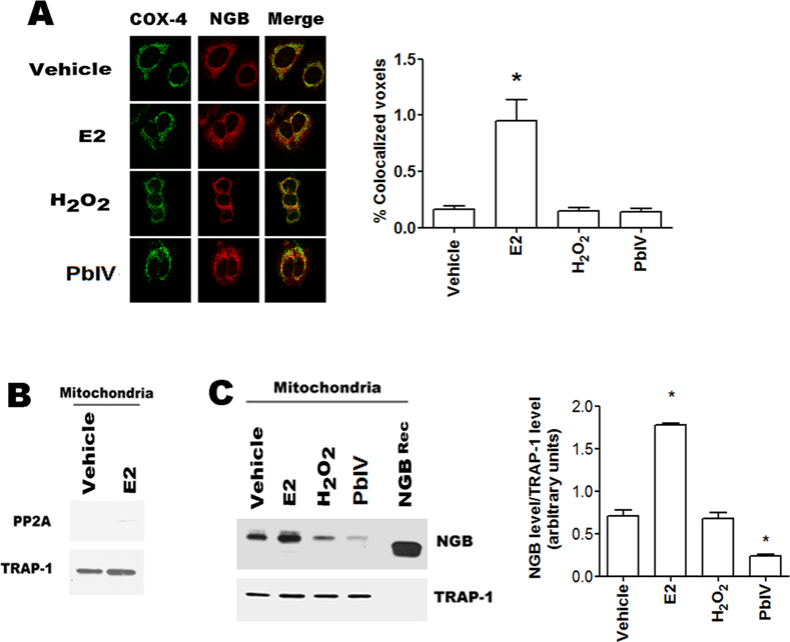
Mitochondrial NGB localization. **(A)** Left, Confocal microscopy analysis of NGB and cytochrome *c* oxidase-4 (COX-4) co-immuno-localization in MCF-7 cells treated for 24h with vehicle and/or E2 (10 nM), H_2_O_2_ (400 μM) and Pb(IV) (200 μM); Right, quantitative analysis of co-localization. Cells were fixed, permeabilized and stained with anti-NGB antibody (red) and co-stained with anti-mitochondrial COX-4 antibody (green) (original magnification x 63). All images are single Z-stack planes and are representative of three independent experiments. **(B)** Western blot analysis of PP2A (cytosolic marker) and TRAP-1 (mitochondrial marker) in mitochondrial fraction of MCF-7 cells treated with either vehicle and/or E2 (10 nM) for 24h. **(C)** Typical Western blot of three independent experiments of NGB expression in mitochondrial fraction of MCF7 cells treated for 24 h with the above reported compounds. Left panel is typical Western blots of three independent experiments. Right panel represents the result of densitometric analyses. The amount of proteins was normalized to the fraction marker protein TRAP-1. Data are means ± SD of three different experiments. P<0.05 was determined with Student t-test vs. vehicle (*).

The NGB localization into the mitochondrial compartment is necessary to act as an anti-apoptotic protein in several cell lines [9, 23, 24]. This prompted us to verify if the selected compounds could modify NGB mitochondrial localization. Confocal microscopy analyses show the co-localization of NGB with the mitochondrial marker COX-4 (cytochrome *c* oxidase-4) (Fig 3A). Although the confocal microscopy allows a purely qualitative analysis, the analysis with the 8.2 IMARIS software demonstrated that only E2 treatment (24h) significantly raises the NGB-COX-4 merged signals (Fig 3A). The increase of the NGB localization at mitochondrial level has been confirmed in isolated mitochondria by using cell fractionation kit. Fig 3B confirms the purity of mitochondrial fraction, in fact PP2A, cytosolic marker is absent; while TRAP-1, mitochondrial marker, is evident. As expected [23], E2 (10 nM; 24 h) increases the mitochondrial NGB content; conversely, H_2_O_2_ treatment (400 μM; 24h) does not modify the NGB mitochondrial localization (Fig 3C). Intriguingly, this more sensitive method demonstrates that Pb(IV) (200 μM, 24 h) treatment determines a significant decrease of the NGB amount in the mitochondrial fraction (Fig 3C).

### Effect of H_2_O_2_ and Pb(IV) on the NGB anti-apoptotic function

**Fig 4:**
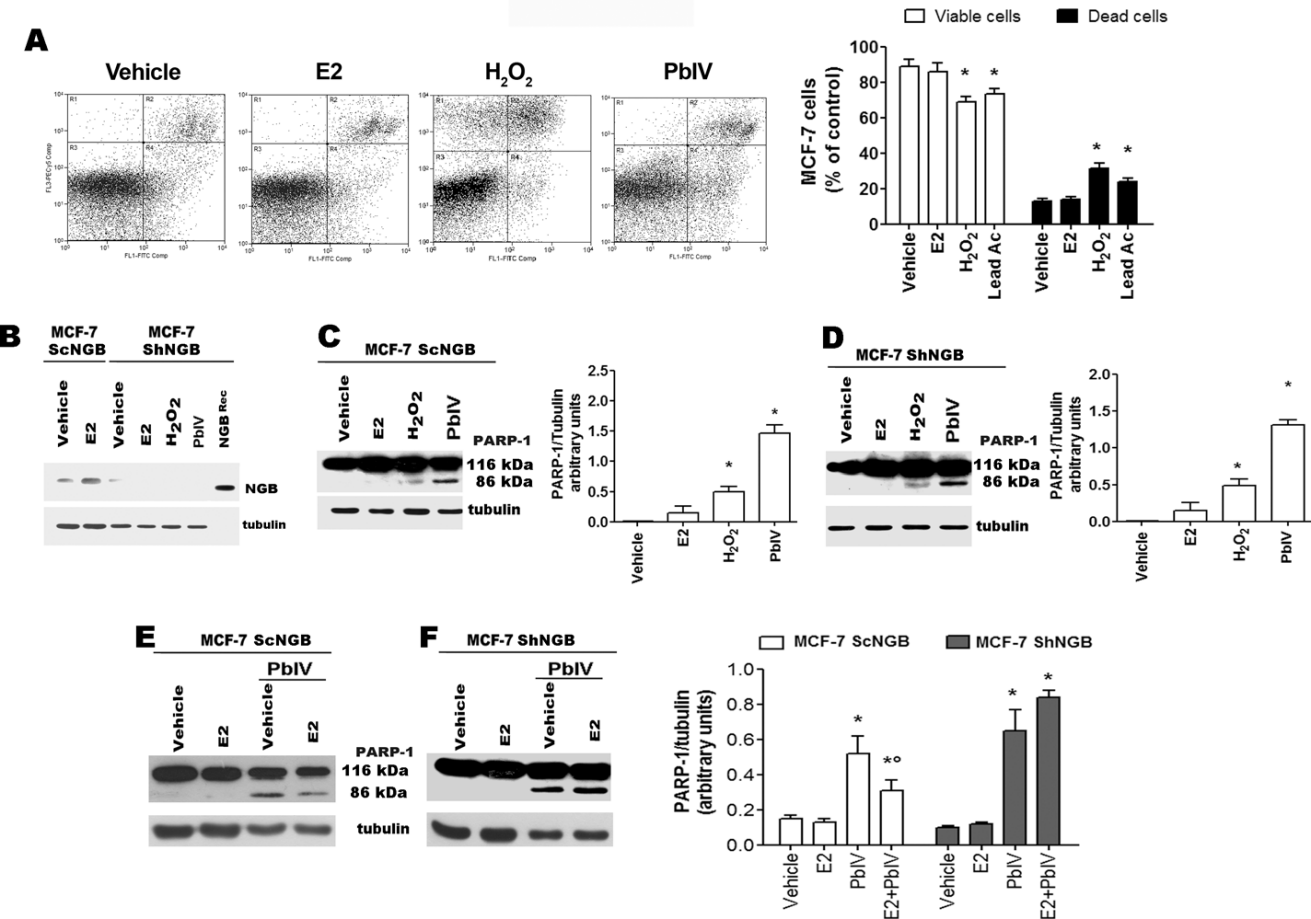
Effect of NGB on H_2_O_2_- and Pb(IV)-induced apoptosis. **(A**) Typical cytograms of vehicle–, E2- (10 nM), H_2_O_2_- (400 μM), and Pb(IV)- (200 μM) treated MCF-7 cells for 24 h (left) and relative analyses (right) obtained from Annexin V-FITC with PI assays. Data of viable (PI and Annexin V-FITC double negative) and dead (Annexin V-FITC positive and PI AnnexinV-FITC double positive) cells are means ± SD of three different experiments. P< 0.05 was calculated with Student’s t test vs vehicle (*). **(B**) Western blot analysis of NGB protein levels performed in vehicle- and E2 (10 nM)- treated control MCF7 cells (ScNGB) and NGB stable silenced cells (ShNGB) treated with selected compounds for 24 h. Typical Western blot representative of three independent experiments. **(C**) Western blot analyses of PARP-1 cleavage in MCF-7 cells infected with scramble RNA (ScNGB MCF-7) treated with above reported compounds for 24h. **(D**) Analysis of protein PARP-1 cleavage in MCF-7 cells infected with silencing NGB shRNA (ShNGB MCF-7) and incubated with E2 (10 nM), H_2_O_2_ (400 μM), and Pb(IV) (200 μM) for 24 h. Western blot analyses of PARP-1 cleavage in MCF-7 cells infected with scramble RNA (ScNGB MCF-7, **(E)**) or with silencing NGB shRNA (ShNGB MCF-7, **(F)**) and treated with E2 (10 nM, 24 h) before the treatment with Pb(IV) (200 μM, 24 h). **(C-F)**, Left panels are typical Western blots of three independent experiments. Right panels represent the result of densitometric analyses. The amount of proteins was normalized by comparison with tubulin levels. Data are means ± SD of three different experiments. P<0.05 was determined with Student t-test vs. vehicle (*) and Pb(IV) (°).

Recently, it has been demonstrated that NGB is an E2 compensatory protein, which up-regulation counteracts apoptosis induced by oxidative stress in several cancer cell lines [23, 24]. This prompted us to evaluate the anti-apoptotic role of NGB in the presence of the selected ROS-inducing compounds. As reported in Fig 4A, H_2_O_2_ and Pb(IV), significantly reduce the percentage of annexin V-FITC/PI double negative MCF-7 cells (viable cells, bottom left panel), and increase the percentage of early apoptotic (annexin V-FITC positive, bottom right panel) and mid-late apoptotic (annexin V-FITC/PI double positive, top right panel) cells. This result has been further confirmed by Western blot analysis in wild type (ScNGB MCF-7) and stable NGB silenced (ShNGB MCF-7) cells (Fig 4B). Surprisingly, although both H_2_O_2_ and Pb(IV) increase NGB level, 24 h after treatment they activate the PARP-1 cleavage, another pro-apoptotic marker, both in the presence and in the absence of NGB (Fig 4C and 4D). This unexpected result lead us to evaluate if E2-induced NGB over-expression in mitochondria could protect MCF-7 cells from Pb(IV)-induced apoptosis. To reach this aim, MCF-7 cells were stimulated with E2 for 24 h before the Pb(IV) treatment. Fig 4E clearly indicate that E2 pre-treatment reduces Pb(IV)-induced PARP cleavage in ScNGB MCF-7, while this effect is completely impaired in ShNGB MCF-7 (Fig 4F).

## Discussion

Here, we investigated the putative role of endogenous level of NGB in breast cancer cells as a stress sensor and as a compensatory protein, which responds to the injuring stimuli inhibiting the trigger of mitochondria-dependent apoptosis. For the first time, we showed that endogenous NGB is a ROS-inducible protein in MCF-7 cells.

Hypoxia is a common feature of solid tumors and the involvement of NGB in the short-term adaptation of cancer cells has been hypothesized [21]. In fact, NGB co-localizes with the hypoxia-inducible metallo-enzyme carbonic anhydrase IX in different human primary tumor specimens [21]. On the other hand, the hypoxia-dependent up-regulation of NGB mRNA has been assessed in lung cancer cells even if no information is available on the protein level [22]. Although NGB is not transcriptionally regulated by HIF1α the major intracellular oxygen sensor [27], this globin has been proposed to be a member of the hypoxia-inducible protein family [4]. Despite this evidence, our results indicate that 2% O_2_, which resembles the median pO_2_ present in breast cancer microenvironment [28], does not up-regulate NGB levels in MCF-7 cells suggesting that NGB is not required for MCF-7 cell adaptation to hypoxic conditions. Of note, myoglobin, which is strongly up-regulated by hypoxia in MCF-7 cells [29], may attend to this function thus suggesting a cell context dependent modulation of NGB from hypoxia

As well hypoxia, oxidative stress is characteristic of both tumor development and cancer cells resistance to antitumor drugs. High ROS levels, as occur in fast proliferating tumor tissues [19], could lead to severe cellular damage and, consequently, to cell death. However, cancer cells established several mechanisms to counteract the oxidative stress-induced apoptosis and, generally, display an antioxidant capacity higher than that of normal cells [19]. Different intracellular pathways could converge to alter the cellular metabolism and to adapt cancer cells to both intrinsic and extrinsic oxidative stress conditions [18]. However, it remains unsolved the question about the possible role of endogenous NGB in non-nervous cancer cells as an oxidative stress sensor; this function requires NGB activation or induction by stressing conditions. Although the PathScan assay and NGB mRNA did not define a unique common pathway linking E2 and stressor-inducing NGB up-regulation, the results reported here clearly demonstrate that in MCF-7 cancer cells the apoptotic inducers modulate the level of NGB. Indeed, cell treatment with H_2_O_2_ and Pb(IV) leads to a rapid increase of intracellular ROS production, and up-regulate NGB protein levels. H_2_O_2_ and Pb(IV) effect on NGB level seems to be mediated by the inhibition of NGB lysosomal degradation and by the activation of translation as demonstrated by cell pre-treatment with Chloroquine and Cicloheximide. Contrarily, MG-132 does not modulate NGB level, but completely impairs H_2_O_2_ and Pb(IV) effect in enhancing NGB level. Recently, a role for MG-132 and Chloroquine in the activation and inhibition, respectively, of autophagy in breast cancer cells has been reported [30, 31] rendering particularly intriguing these results. Indeed, our results seem suggest that the autophagic process is involved in H_2_O_2_- and Pb(IV)-NGB accumulation breast cancer cells. Finally, the involvement of AKT, SMAD2, SAPK/JNK, survivin, and protein stability, evidenced in this study, could converge in ROS-inducing pathways (e.g., Nuclear factor erythroid-derived 2, NRF2) to increase NGB levels and, ultimately, to cancer cell survival. Although studies more detailed are requested to define the ROS-induced pathways and their functional outcomes, the data reported in this study enlarge the physiological role of NGB in breast cancer cells pointing to its up-regulation as possible ROS sensor as reported in brain-derived cells [2–4, 6, 11, 17]. In particular, in human and rat pheochromocytoma PC12 cell lines, the H_2_O_2_ treatment increases the level of transiently transfected NGB and induces a conformational change of NGB allowing the globin recruitment at the plasma membrane lipid rafts where it acts as a guanine-dinucleotide dissociation inhibitor suppressing the Gαs activity and leading to neurons protection against apoptosis [17]. However, data reported here unexpectedly indicate that the increased level of NGB induced by H_2_O_2_ and Pb(IV) is not sufficient to counteract the ability of these substances to induce the apoptotic death in MCF-7. This result has been further confirmed by NGB silencing experiments in which both H_2_O_2_ and Pb(IV) still activate the PARP-1 cleavage, an apoptotic marker in MCF-7 cells. However, increasing NGB levels by 24 h E2 treatment reduces Pb(IV) activation of PARP-1 cleavage. This result is in line with the E2 protective effect against H_2_O_2_–induced apoptosis previously reported [23–24] strongly confirming the anti-apoptotic role of NGB.

Remarkably, E2-induced NGB up-regulation exerts anti-apoptotic function directly at the mitochondrial compartment by interacting with cytochrome *c* and impairing its release to cytosol and the consequent activation of the intrinsic apoptotic pathway upon oxidative stress injury [7, 23]. Present data indicate that in MCF-7 cells, only the E2 treatment almost doubles the NGB amount in the mitochondrial fraction, whereas H_2_O_2_ and Pb(IV), which increase NGB level in the whole cell, do not affect the mitochondrial protein amount. All together, these data indicate that the increase of intracellular NGB levels induced by H_2_O_2_ and Pb(IV) is not sufficient to reset the intrinsic apoptotic pathway, which requires the re-allocation of NGB into mitochondria. In line with this idea, Yu and coworkers [6] demonstrated that in primary mouse cortical neurons NGB mitochondrial localization increased after pathological oxygen-glucose deprivation conditions conferring neuroprotection. Although it could be possible that only the high NGB levels obtained by transfection with NGB-encoding plasmid allow the NGB recruitment on lipid rafts reported in neurons after oxidative stress [17], the possibility that E2, oxygen-glucose deprivation, and oxidative stress signaling could induce different NGB conformation that affect the protein translocation into mitochondria should be taken into account.

An opposite function has been attributed to NGB in hepatocarcinoma cells. In this cell line, NGB acts as a mediator between oxygen/ROS signals and the cytosolic signaling cascade that regulates the cell proliferation [20]. Unfortunately, also this evidence has been obtained in cells transiently transfected with the NGB-encoding plasmid which induces high non-physiological protein levels that could be necessary for NGB involvement in intracellular signaling cascade [20]. However, the involvement of NGB in cancer cell proliferation has not been confirmed by MCF-7 cell growth curves, which result similar to the control even when NGB was stably silenced [23]. These results indicate that the over-expression of NGB does not fully represent the physiological behavior of NGB.

In conclusion, NGB level could be considered as a sensor of ROS being up-regulated by ROS (i.e., H_2_O_2_) and by ROS-inducing substances (i.e., Pb(IV)), whereas its function as an anti-apoptotic protein is strictly linked to its level and intracellular localization. However, oxidative conditions increase the heme-Fe-based NGB reactivity by formation of the labile Cys46-Cys55 disulfide bond and of the Tyr44/His64/heme propionate interaction [32, 33]. Consequently, ROS and ROS-generating compounds induce high level of oxidized NGB that increase, for example, NGB activity as free radical scavenger [34] supporting the role of NGB as a compensatory protein in breast cancer cells. Although further studies in breast cancer cells are needed to identify up-stream and down-stream NGB regulating pathways and the intracellular NGB trafficking, present data might lead to a new direction in understanding NGB function cancer and neuroprotection opening new avenues for the therapeutic intervention based on the development of inhibitors impairing NGB-dependent cell protection.

## Supporting Information

S1 Fig Map position of p-AKT, p-SAPK/JNK, p-SMAD2, PARP-1 and survivin specific antibody in the PathScan® Stress and Apoptosis Signaling Antibody ArrayKit. (TIF)

## Abbreviations

Act: actinomycin D
BSA: bovine serum albumin
COX-4: anti-mitochondrial cytochrome c oxidase-4
DCFH-DA: 2’,7’-dichlorofluorescin diacetate
DMEM: Dulbecco’s modified Eagle medium
E2: 17β-estradiol
IPTG: isopropyl-D thiogalactopyranoside
NGB: human neuroglobin
PARP-1: poly(ADP ribose) polymerase 1
Pb(IV): lead(IV) acetate
PI: propidium iodide
PP2A: cytosolic Protein Phosphatase 2A
control RNA: ScNGB
shRNA: short hairpin RNA
Tx: paclitaxel (taxol
ROS: reactive oxygen species
TRAP-1: TNF-receptor associated protein-1
