# Supplementary figures and images for: Neuroglobin in Breast Cancer Cells: Effect of Hypoxia and Oxidative Stress on Protein Level, Localization, and Anti-Apoptotic Function
Source: PLoS One. 2016 May 5;11(5):e0154959. doi: 10.1371/journal.pone.0154959 (PMC4858147; doi:10.1371/journal.pone.0154959)

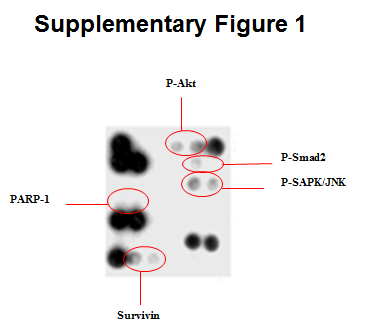

Supplement: S1 Fig — (TIF) [file pone.0154959.s001.tif]
